# Maximizing Nanoscale Disorder in Block Copolymers for Orientation‐Independent SERS Platform Toward Non‐Invasive Diagnostics

**DOI:** 10.1002/advs.76631

**Published:** 2026-07-14

**Authors:** Jin Man Kim, Wonsik Kim, Wansun Kim, Yeon‐Hee Kim, Samjin Choi, Jang Hwan Kim, Hyeong Min Jin

**Affiliations:** ^1^ Department of Organic Materials Engineering Chungnam National University Daejeon Republic of Korea; ^2^ Department of Materials Science and Engineering Chungnam National University Daejeon Republic of Korea; ^3^ Department of Energy Systems Research Ajou University Suwon Republic of Korea; ^4^ Department of Biomedical Engineering College of Medicine, Kyung Hee University Seoul Republic of Korea; ^5^ Department of Obstetrics and Gynecology College of Medicine The Catholic University of Korea Seoul Republic of Korea; ^6^ Department of Materials Science and Engineering Ajou University Suwon Republic of Korea

**Keywords:** biosensor, block copolymer, nanopatterning, plasmonic, SERS

## Abstract

The development of high‐performance optical molecular diagnostic platforms requires the precise engineering of light–matter interactions to ensure quantitative accuracy across diverse sensing environments. While high‐density nanogap architectures based on block copolymer‐derived lamellar patterns provide exceptional sensing performance, their inherent long‐range orientational order induces polarization‐dependent responses that compromise quantitative reliability. Here, we introduce controlled randomness into vertically aligned lamellae to induce optical isotropy while strictly preserving nanoscale periodicity. Inspired by natural ridge‐like architectures, grain‐size regulation generates stochastically oriented domains with short‐range orientational correlation. Engineering orientational disorder through grain‐size‐mediated diversity suppresses long‐range anisotropy while upholding the integrity of the periodic framework. Quantitative assessments of structural entropy and numerical simulations of localized hot‐spots provide in‐depth insights into the underlying mechanism. The resulting stochastic architectures exhibit SERS responses that are insensitive to polarization and incident direction while maintaining spatially uniform signal reproducibility. The statistically robust SERS platform enables high‐fidelity clinical diagnostics, demonstrated through the metabolic profiling of urine from pregnant women and AI‐driven predictive modeling, establishing a universal basis for quantitative molecular sensing.

## Graphical abstract

Maximizing Nanoscale Disorder in Block Copolymers for Orientation‐Independent SERS Platform toward Non‐Invasive Diagnostics is a nature‐inspired strategy that engineers controlled randomness within block copolymer lamellae to achieve optical isotropy without compromising nanoscale periodicity. By regulating grain size to induce orientational disorder, this platform provides polarization‐independent SERS responses, enabling robust, AI‐driven metabolic profiling for high‐fidelity, non‐invasive clinical diagnostics.

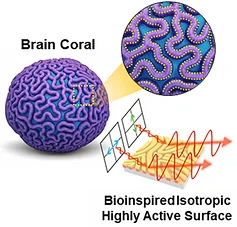

## 1 Introduction

Nature selectively adopts complex and non‐regular structural motifs as effective functional strategies, rather than strictly ordered architectures. In spatially confined environments, irregular geometries can enhance efficiency by maximizing interfacial area and enabling multidirectional interaction pathways [1, 2, 3, 4, 5]. For example, deep‐sea brain corals exhibit maze‐like wrinkled surfaces that enhance light–matter interactions [6, 7], while plant leaves contain irregular porous networks that optimize mass‐transfer efficiency [2, 3]. These biological architectures demonstrate that intrinsic structural fluctuations, when appropriately constrained, can be transformed into functional morphologies that outperform perfectly ordered systems [8, 9]. Accordingly, efficiency‐driven design principles observed in nature motivate engineered architectures for nanoscale optical and sensing platforms, where surface‐mediated interactions are highly sensitive to geometry and orientation [10, 11, 12].

Among such nanoscale sensing platforms governed by surface‐mediated light–matter interactions, surface‐enhanced Raman spectroscopy (SERS) amplifies intrinsically weak Raman signals through localized surface plasmon resonance (LSPR) excited in metallic nanostructures [11, 13]. While high enhancement factors (EF) have been widely reported, achieving high signal reproducibility over large areas remains a central challenge for practical SERS applications. Importantly, high‐performance SERS substrates must satisfy two key structural requirements: (i) a narrow distribution of nanogap spacing to ensure uniform local enhancement [14, 15, 16], and (ii) an orientation‐independent, isotropic optical response that is insensitive to measurement direction or polarization [17, 18]. These requirements indicate that neither perfectly ordered architectures nor indiscriminately random structures are optimal [13, 14, 18]. Instead, SERS substrates demand architectures that preserve uniform nanoscale length scales while mitigating long‐range orientational anisotropy [14, 15, 17].

Molecular self‐assembly provides a physical framework that naturally satisfies these structural requirements [19, 20]. In self‐assembled systems, building blocks reorganize under weak and reversible interactions while exploring complex energy landscapes, resulting in morphologies that exhibit local order combined with global complexity. Because characteristic length scales are defined by thermodynamic interactions, local structural uniformity can be maintained, while variations in domain orientation emerge through controlled kinetic pathways [21, 22]. Importantly, these processes remain deterministic, allowing the degree of structural complexity to be reproducibly tuned through external parameters [21, 23]. Such features make self‐assembly particularly well suited for SERS substrates, where uniform hot‐spot spacing must coexist with suppressed macroscopic anisotropy [19, 20]. Recent perspectives on intelligent BCP self‐assembly have further highlighted its capability for scalable, reproducible, and large‐area nanopatterning, underscoring its relevance as a manufacturing platform for next‐generation device applications [24].

Among various self‐assembled architectures, lamellar block copolymer (BCP) morphologies offer a particularly advantageous structural platform for reproducible SERS substrates. Vertically aligned lamellae possess extended and continuous interfacial geometries, which naturally support dense line‐type plasmonic hot‐spot networks with high effective hot‐spot density [25, 26, 27]. At the same time, the intrinsic nanoscale periodicity of lamellar domains defines uniform nanogap spacing, directly satisfying a key requirement for reproducible SERS enhancement [25, 27]. However, conventional lamellar patterns typically exhibit strong long‐range orientational order, which induces structural anisotropy and leads to polarization‐dependent optical responses that compromise signal reproducibility [28, 29].

This limitation is particularly important for line‐type SERS platforms. Unlike point‐like nanogap architectures, line‐type lamellar structures provide extended SERS‐active interfaces that can be continuously sampled within the laser excitation area. However, when these line‐type interfaces are aligned over long ranges, the resulting optical response becomes highly dependent on the relative orientation between the nanogap direction and the incident polarization. Therefore, achieving isotropy in a line‐type framework requires a structural design that does not simply randomize the entire pattern but selectively suppresses long‐range orientational anisotropy while preserving nanoscale periodicity and continuous nanogap interfaces. This requirement provides the design rationale for the present study.

Here, we overcome the optical anisotropy inherent in highly oriented structures by introducing controlled randomness into the local orientation of vertically aligned BCP lamellae patterns while preserving their intrinsic nanoscale periodicity. Inspired by natural ridge‐like architectures that combine uniform spacing with multidirectional orientation, we regulate the grain size of the template to generate stochastically oriented domains with short‐range orientational correlation. This strategy effectively decouples orientational disorder from nanoscale length‐scale uniformity: the lamellar half‐pitch constrains the nanogap length scale, whereas grain‐size‐controlled orientation diversity suppresses long‐range anisotropy at the substrate scale. As a result, these disordered architectures exhibit SERS responses that are insensitive to polarization and incident direction with reduced point‐to‐point variability. Motivated by this large‐area uniformity and statistical stability, we extend the platform to a clinically relevant setting by acquiring urine SERS spectra from pregnant women for metabolic biomarker profiling, establishing a robust basis for quantitative readout and artificial intelligence (AI)‐driven diagnostic modeling.

## 2 Results and Discussion

### 2.1 Bioinspired Entropy‐Controlled Lamellar Templates for Isotropic Plasmonic Interfaces

Skeletal structures of brain corals exhibit evolutionarily optimized light‐scattering characteristics, utilizing intrinsic porosity and irregular microarchitecture to maximize photosynthetic efficiency. This complex geometry promotes isotropic angular distribution and deep light penetration, establishing a homogeneous photic environment within the tissue [7, 30]. Inspired by these naturally optimized architectures, we emulate a comparable degree of structural randomness in a nanoscale fingerprint template to develop an optically efficient and direction‐robust photonic platform (Figure 1a). To translate this bio‐inspired concept into a functional nanostructure, we used the self‐assembly of a polystyrene‐block‐poly(methyl methacrylate) (PS‐*b*‐PMMA). Precise tailoring of self‐assembly kinetics enables modulation of domain ordering, ranging from highly aligned to randomly oriented configurations [21, 31]. Notably, the resulting small‐grain architecture, composed of multiple domains with various orientations, effectively prevents the macroscopic anisotropy typically associated with long‐range ordering. This spatially distributed fine‐grained morphology serves as an ideal direction‐independent photonic platform, offering both high effective interfaces and isotropic responses essential for reliable surface‐mediated optical applications (Figure 1b).

**FIGURE 1:**
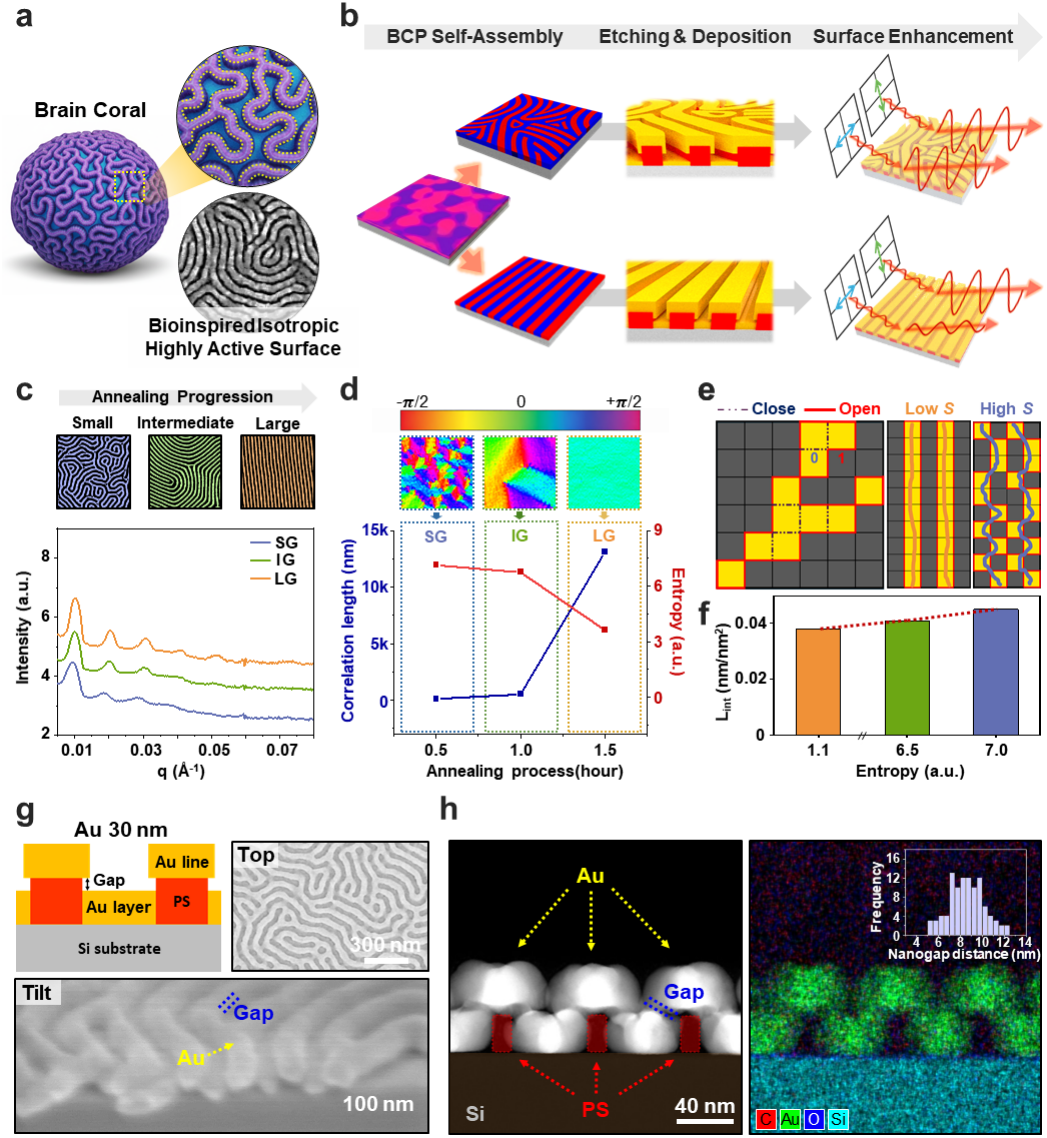
(a) Brain coral skeletal/tissue structures and biomimetic random‐orientation vertical lamellae inducing optical isotropy. (b) Schematic illustration of the fabrication of SERS substrates with controlled grain sizes. (c) Grain‐size control by SVA and corresponding GI‐SAXS profiles. (d) Local orientation maps (top) and extracted orientational correlation length (*ξ*) and orientational entropy (*S*) versus SVA time (bottom). (e) Pixel‐based “open/closed” boundary model for accessible interface quantification. (f) Accessible interfacial‐length density (*L_int_*) correlated with entropy. (g) Cross‐sectional schematic representation of an Au plasmonic nanogap array and representative SEM images (t = 30 nm). (h) Cross‐sectional TEM and EDS mapping images of the Au‐defined nanogap architecture at t = 30 nm, with an inset gap‐width distribution histogram of 8.5 ± 1.56 nm from 100 measured gap positions.

Strategic modulation of structural randomness in BCP lamellar patterns was achieved by precisely regulating grain size via solvent vapor annealing (SVA) time. Systematic extension of annealing time promotes lamellar grain growth, modulating both the orientational correlation and the degree of disorder to yield small‐grain (SG), intermediate‐grain (IG), and large‐grain (LG) lamellar patterns, respectively (Figure 1c, **top**). To quantify the lateral periodicity and the degree of long‐range order in the patterns, grazing‐incidence small‐angle X‐ray scattering (GI‐SAXS) measurements were performed. The GI‐SAXS profiles yielded a consistent lamellar period (*L_0_*) across grain‐size conditions. However, analysis of the full width at half maximum (FWHM) demonstrated that the peaks became progressively sharper and narrower from SG to LG (Table 1). This clearly suggests that lamellar patterns with larger grains exhibit higher in‐plane long‐range order, corresponding to a longer correlation length (Figure 1c, **bottom**) [22, 32]. The peak narrowing also indicates reduced nanoscale positional and orientational disorder, consistent with more coherent lamellar interfaces. These findings quantitatively confirm that the extension of SVA time drives grain growth and expands locally ordered regions to the microscale [33, 34, 35].

**TABLE 1:** Quantitative structural parameters of BCP templates derived from GI‐SAXS.

	SG	IG	LG
q* (Ǻ^−1^)	0.00939	0.01001	0.01001
*L_0_* (nm)	66.9	62.7	62.7
FWHM (Ǻ^−1^)	0.00348	0.00313	0.00307

In‐depth quantitative analysis was performed to establish a rigorous physical framework for the controlled disorder observed in the self‐assembled templates [36, 37]. Scanning electron microscopy (SEM) images were converted into pixel‐wise 2D orientation maps using structure tensor analysis, providing local director fields across the entire pattern (Figure 1d, **top**). The orientational correlation length (*ξ*) increases progressively from SG to LG [38, 39, 40]. A shorter *ξ* in SG signifies rapid decorrelation of local orientations, supporting a more isotropic, polarization‐independent response [41, 42]. Briefly, grayscale‐normalized and mean‐subtracted SEM images across representative regions were processed via 2D fast Fourier transform (FFT). The azimuthal intensity distribution (***p***_***i***_) was extracted at the dominant structural frequency (***q*** ^*^) using 1° angular binning (0°–180°), which was then applied to the Shannon entropy formula to rigorously quantify the directional disorder. Crucially, the structural complexity and local fluctuation of the patterns were quantified through the orientational entropy (*S*) [43]. The SG state exhibits a quasi‐isotropic orientation distribution, corresponding to a high‐entropy state, whereas the LG state shows pronounced directional bias, resulting in lower orientational entropy (Figure 1d, **bottom**) [44].

Optically addressable interactions in lamellar templates occur primarily at interfaces exposed to the external environment [45, 46]. Evaluation of the available interface utilized a pixel‐based occupancy model distinguishing between “open” boundaries (value = 1), which are accessible for external interaction, and “closed” regions (value = 0) (Figure 1e) [36, 47]. The analysis reveals a direct positive correlation between orientational entropy and the open interfacial length (*L*_int_), confirming that high‐entropy architectures provide significantly larger optically accessible interfaces (Figure 1f) [48, 49]. Consequently, we define the small‐grain (SG) morphology as a high‐entropy architecture (HE), characterized by short‐range orientational correlation and maximized optically accessible interfaces. In this framework, the intermediate‐grain (IG) and large‐grain (LG) morphologies are classified as medium‐entropy (ME) and low‐entropy architectures (LE), respectively, reflecting progressively increased orientational correlation and reduced accessible interfacial length [50, 51].

With the high‐entropy morphology established as a structural base, the fabrication was extended to incorporate metallic functionality for optical applications. UV crosslinking and selective etching were carried out to preserve the PS framework as a template. Gold (Au) was then deposited via electron‐beam evaporation, transforming the high‐entropy dielectric template into a plasmonically active platform. Due to the directional nature of deposition, Au forms a continuous line on the PS tops and the underlying Au layer, whereas limited sidewall coverage effectively leaves nanogaps (*g*) at the junctions that serve as near‐field coupling sites (Figure 1g). Cross‐sectional transmission electron microscopy (TEM) analysis clearly revealed the formation of Au nanogaps at the junction between the Au‐coated PS features and the underlying Au layer. To quantitatively evaluate the nanogap geometry, a gap‐width distribution histogram was obtained from 100 measured gap positions. The resulting analysis yielded an average gap width of 8.5 ± 1.56 nm, confirming that the Au deposition process reproducibly forms nanoscale junction gaps predominantly around the sub‐10 nm regime. Furthermore, energy‐dispersive X‐ray spectroscopy (EDS) elemental mapping confirmed the distinct spatial distribution of Au, C, and Si, validating that the Au‐coated PS sidewalls and the underlying Au layer are precisely organized to form the nanogap architecture (Figure 1h). Collectively, these structural and compositional analyses demonstrate the successful formation of Au‐defined nanogap arrays that provide geometrically confined regions for near‐field enhancement.

### 2.2 Entropy‐Driven Isotropic, Uniform, and Scalable SERS Performance

The Au thickness (*t*) was prioritized as the primary design parameter to maximize the electromagnetic field enhancement within the established nanogaps (Figure S1). Given that *t* directly governs the nanogap geometry and the resulting plasmonic coupling, its optimization is critical to linking template‐level structural metrics to SERS performance [19, 52, 53]. Raman spectra were collected using 10^−3^ M thiophenol as a standard probe molecule (Figure 2a). The corresponding intensity analysis identified 30 nm as the optimal thickness, yielding high SERS intensity with a low relative standard deviation (RSD) (Figure 2b). Observations showed that as *t* increased beyond ∼60 nm, the nanogaps were nearly eliminated, which consequently suppressed the formation of hot‐spots (Figure S2). With the Au thickness fixed at the optimized 30 nm, the SERS responses of different templates were compared to isolate the specific impact of structural entropy on plasmonic performance. The HE exhibited an approximately 1.5‐fold higher SERS enhancement than the LE counterpart (Figure 2c). This enhancement is consistent with the significantly greater open interfacial length (*L*_int_) of the high‐entropy state, which increases the areal density of Au‐defined nanogaps sampled within the laser excitation area. It is notable that this maximization of SERS performance occurs despite the inherent microstructural limitations of the high‐entropy regime. HE structure inevitably accompanies line‐end defects alongside enhanced lamellar fluctuation. Because the effective SERS hot‐spots in our system are formed exclusively at the vertical sidewalls of the lamellae, thermodynamic aggregation at these defect sites and the resulting interfacial curvature act as hindering factors that degrade gap quality and reduce the effective footprint available for active sidewalls. Nevertheless, the maximized SERS performance is observed in the HE structure because the geometric expansion of the active sidewall length, driven by severe continuous fluctuation, overwhelmingly dominates the hot‐spot attenuation caused by the defects. The structural isotropy of the HE was evaluated through the rotation‐angle dependence of the SERS response (Figure 2d). Notably, the HE template maintains a consistently high intensity across all rotation angles, exhibiting a nearly circular radial envelope in the polar plot. The observed circular symmetry demonstrates an independence from the incident light polarization, whereas LE templates exhibit pronounced angle‐dependent fluctuations due to their directional ordering. To further quantify spatial uniformity, polarized optical microscopy (POM) analysis was conducted, and Raman mapping was performed over a 50 µm × 50 µm area (Figure 2e and Figure S3). The HE surface appeared visually homogeneous, yielding the highest mean intensity with the narrowest point‐to‐point distribution and a RSD of 8.3% (Figure 2f), confirming the spatially uniform SERS signal reproducibility of the HE substrate at the local mapping scale. Finally, wafer‐scale scalability was demonstrated by fabricating randomly oriented lamellar structures over an entire 4‐inch wafer (Figure 2g). SERS measurements at 15 positions across the wafer yielded an RSD of 7.2% for the thiophenol band at 1073 cm^−^^1^, confirming uniform enhancement over centimeter‐scale areas (Figure 2h). In addition, repeated measurements on the same substrate over two months showed no appreciable loss of SERS intensity (Figure 2i), supporting large‐area reproducibility and short‐term temporal stability for practical deployment.

**FIGURE 2:**
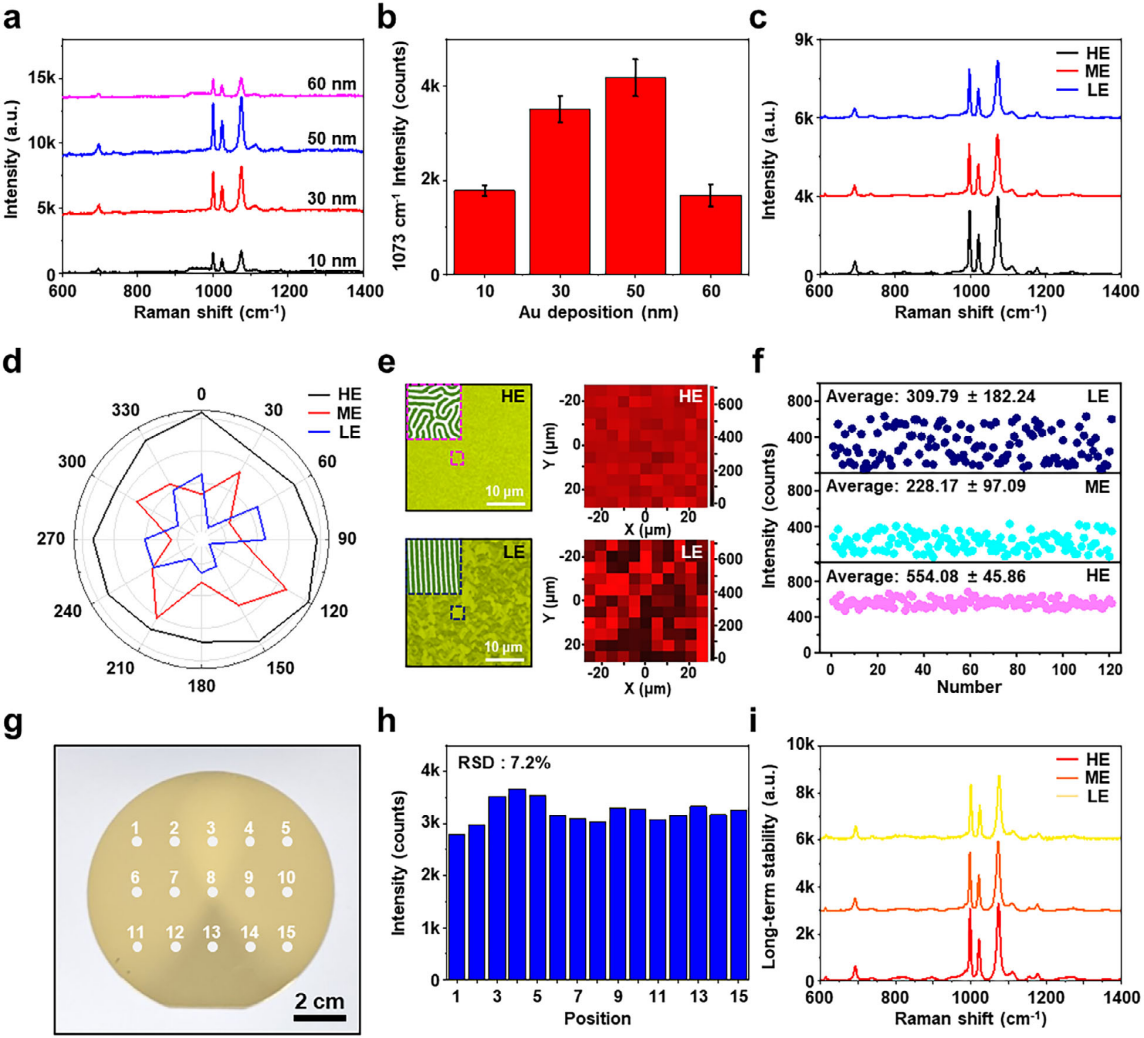
(a) Measured SERS spectra of 1 mM thiophenol at four different Au thicknesses (10, 30, 50, and 60 nm). (b) Thickness‐dependent SERS intensity at 1073 cm^−1^ of 1 mM thiophenol for Au thicknesses of 10, 30, 50, and 60 nm. (c) Measured SERS spectra of 1 mM thiophenol at three different entropy levels (HE, ME, and LE). (d) SERS intensity of thiophenol at 1073 cm^−1^ measured as the HE substrate was rotated through 360°. (e) Polarized optical microscopy (POM) images and corresponding Raman intensity maps comparing high‐entropy (HE) and low‐entropy (LE) architectures. (f) SERS intensity at 1073 cm^−1^ measured at 121 points within the maps. (g) Fabrication of HE SERS substrate over a 4‐inch wafer scale. (h) Distribution of thiophenol SERS intensity at 1073 cm^−1^ measured at 15 locations on the wafer scale HE SERS substrate. (i) Long‐term stability of the SERS intensity evaluated over a two‐month period under ambient conditions.

### 2.3 Entropy‐Dependent Plasmonic Responses and Excitation Wavelength Optimization

To rationalize the observed uniform enhancement and identify the optimal excitation conditions for HE, the wavelength‐dependent plasmonic response was evaluated using a finite element analysis (FEA) model. The simulated electric‐field distribution shows pronounced localized hot‐spots within the nanogap between the Au line on the PS top and the underlying Au layer with the strongest enhancement at an excitation wavelength of 785 nm (Figure 3a). Notably, HE produces stronger field enhancement than LE, while both exhibit a maximum at 785 nm (Figure 3b and Figure S4).

**FIGURE 3:**
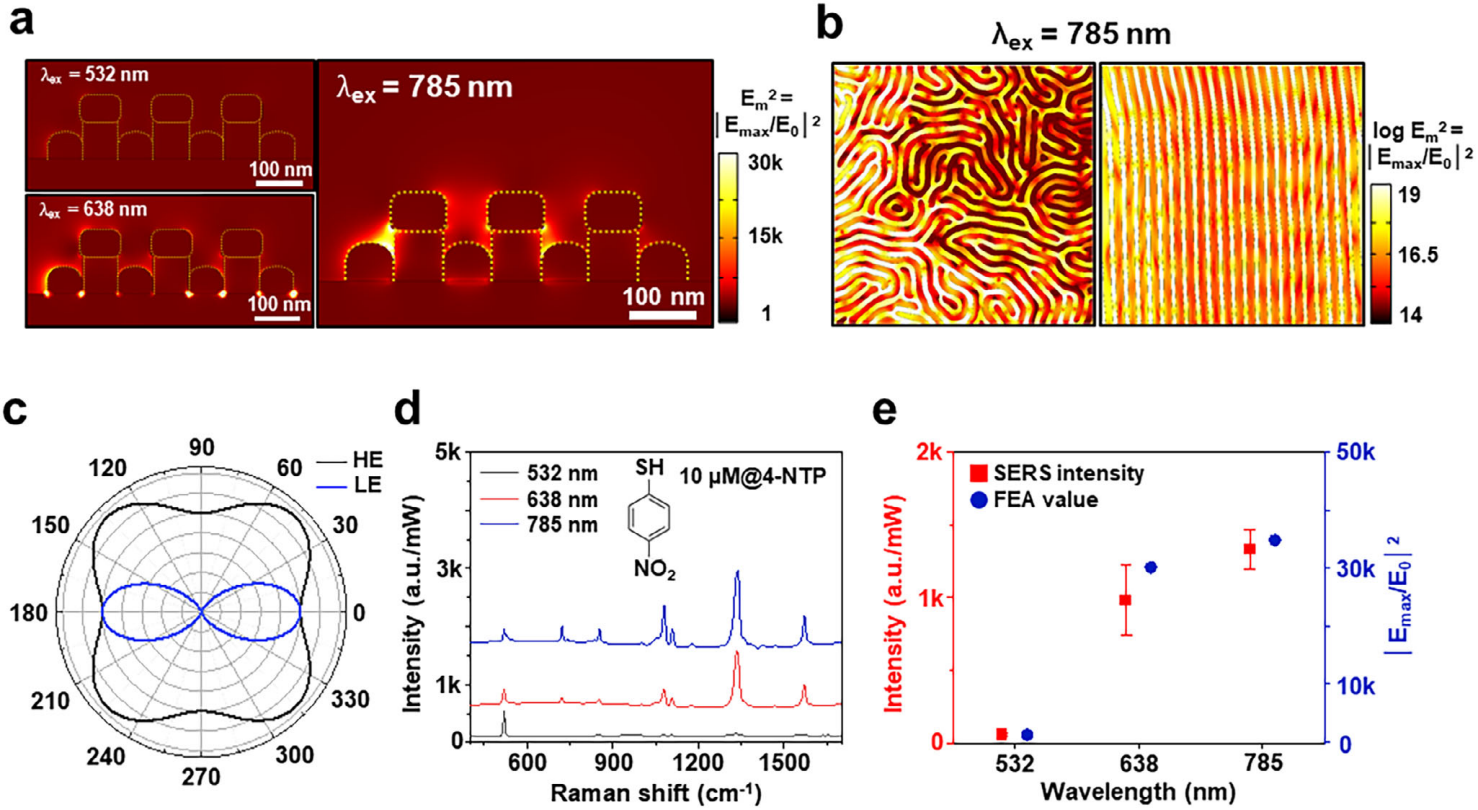
(a) Finite element analysis (FEA)‐simulated cross‐sectional electric field distributions of the proposed model and (b) FEA‐simulated top‐view electric field distributions of 785 nm wavelength. (c) Polarization‐dependent field enhancement simulated for HE and LE. (d) Experimentally measured SERS spectra of 10 µM 4‐NTP at three different excitation wavelengths. (e) Comparative analysis of simulated electric field enhancement and experimental SERS intensity at the 1338 cm^−^^1^ Raman peak of 4‐NTP for each excitation wavelength.

Polarization sensitivity was also examined to clarify how structural alignment affects the plasmonic response. Consistent with the polarization anisotropy observed experimentally for LE (Figure 2d), the simulated field enhancement is strongly modulated upon rotating the incident polarization, yielding the characteristic response of a lamellar pattern. This behavior arises from the one‐dimensionally aligned Au nanogap geometry, where plasmonic coupling and local field enhancement are preferentially excited for specific polarization directions. Accordingly, long‐range‐ordered substrates such as LE can exhibit pronounced polarization‐dependent responses, leading to a macroscopic anisotropic response at the substrate scale. In contrast, in HE, the grain size is sufficiently small relative to the laser beam size (<0.84 µm) that domains with diverse orientations are averaged within the excitation area, resulting in a largely polarization‐ and direction‐insensitive response (Figure 3c).

These predictions were validated experimentally using wavelength‐ and concentration‐dependent SERS measurements on HE with 4‐nitrothiophenol (4‐NTP) as a probe molecule. SERS spectra acquired at 532, 638, and 785 nm showed the strongest signal at 785 nm (Figure 3d). The incident‐power‐normalized intensity followed the same wavelength‐dependent trend as the FEA‐derived field‐enhancement metric, |*E*_max_ */E_0_* |^2^ (Figure 3e), confirming that plasmonic coupling is most efficiently excited at 785 nm. Based on this optimized excitation condition, the analytical sensitivity of the HE substrate was further evaluated using concentration‐dependent 4‐NTP measurements. The SERS intensity at 1338 cm^−^^1^ showed a linear response from 10^−^^8^ to 10^−^^5^ M, yielding a LOD of 67.3 nM and an EF of ∼2.1 × 10^5^ (Figures S5 and S6).

Although several state‐of‐the‐art SERS substrates have reported higher absolute EFs through highly optimized 3D, hierarchical, lithographically defined, or strongly coupled plasmonic nanogap architectures (EF ≈ 10^7^–10^10^) [54, 55, 56], these platforms typically require cost‐intensive serial fabrication (e.g., electron‐beam lithography, atomic‐layer‐deposition‐defined gaps, or focused‐ion‐beam milling) or provide limited large‐area uniformity data. In contrast, the present HE substrate emphasizes a balanced performance profile that integrates sufficient enhancement, low signal variation, wafer‐scale reproducibility, and reduced sensitivity to polarization and incident direction. The 10^5^‐level EF (∼2.1 × 10^5^) is accompanied by sub‐10% signal variation (wafer‐scale spot‐to‐spot RSD = 7.2%), which is comparable to other scalable self‐assembly‐ or template‐based SERS platforms reported at similar EF levels, such as laser‐MBE‐grown Ag nanoislands (EF ≈ 1.2 × 10^5^, RSD ≈ 7.8%) [57] and cicada‐wing‐templated flexible substrates (EF ≈ 4.2 × 10^5^, RSD ≈ 2.8%–7.3%) [58]. Notably, compared with the previously reported BCP‐nanopost Au nanogap array (EF ≈ 10^4^, RSD ≈ 12.3%) [19], the present HE architecture achieves both higher enhancement and markedly improved reproducibility, while additionally providing polarization‐independent response in a line‐type lamellar framework. A detailed quantitative comparison is provided in Table S1. These results highlight the practical advantage of the high‐entropy BCP lamellar architecture: reproducible and optically isotropic SERS performance achieved through a simple and scalable self‐assembly‐based process.

### 2.4 AI‐Enabled SERS for Early Preeclampsia Screening

Building on the polarization‐insensitive and spatially uniform SERS response of the HE, we evaluated its utility for data‐driven disease classification. As a clinically relevant test case, we targeted early‐stage diagnosis of preeclampsia, a pregnancy‐specific hypertensive disorder that remains challenging to detect during early gestation. Using urine samples collected from pregnant women (*n* = 11 per group), we implemented an AI‐assisted SERS classification workflow to distinguish healthy controls from patients with preeclampsia (Figure 4a). Urine SERS spectra acquired on HE exhibited well‐defined Raman bands with high signal‐to‐noise ratios and excellent reproducibility across repeated measurements (Figure 4b and Figure S7). This reproducibility is consistent with spatially uniform plasmonic activity and thereby supports quantitative analysis and machine‐learning‐based modeling. The spectra contained characteristic vibrational features of human urine [59, 60], while univariate comparisons did not reveal significant group‐wise differences in protein‐associated bands previously linked to preeclampsia [61], including the amide III (1200–1300 cm^−1^) and amide I (∼1650 cm^−1^) regions (Figure 4c).

**FIGURE 4:**
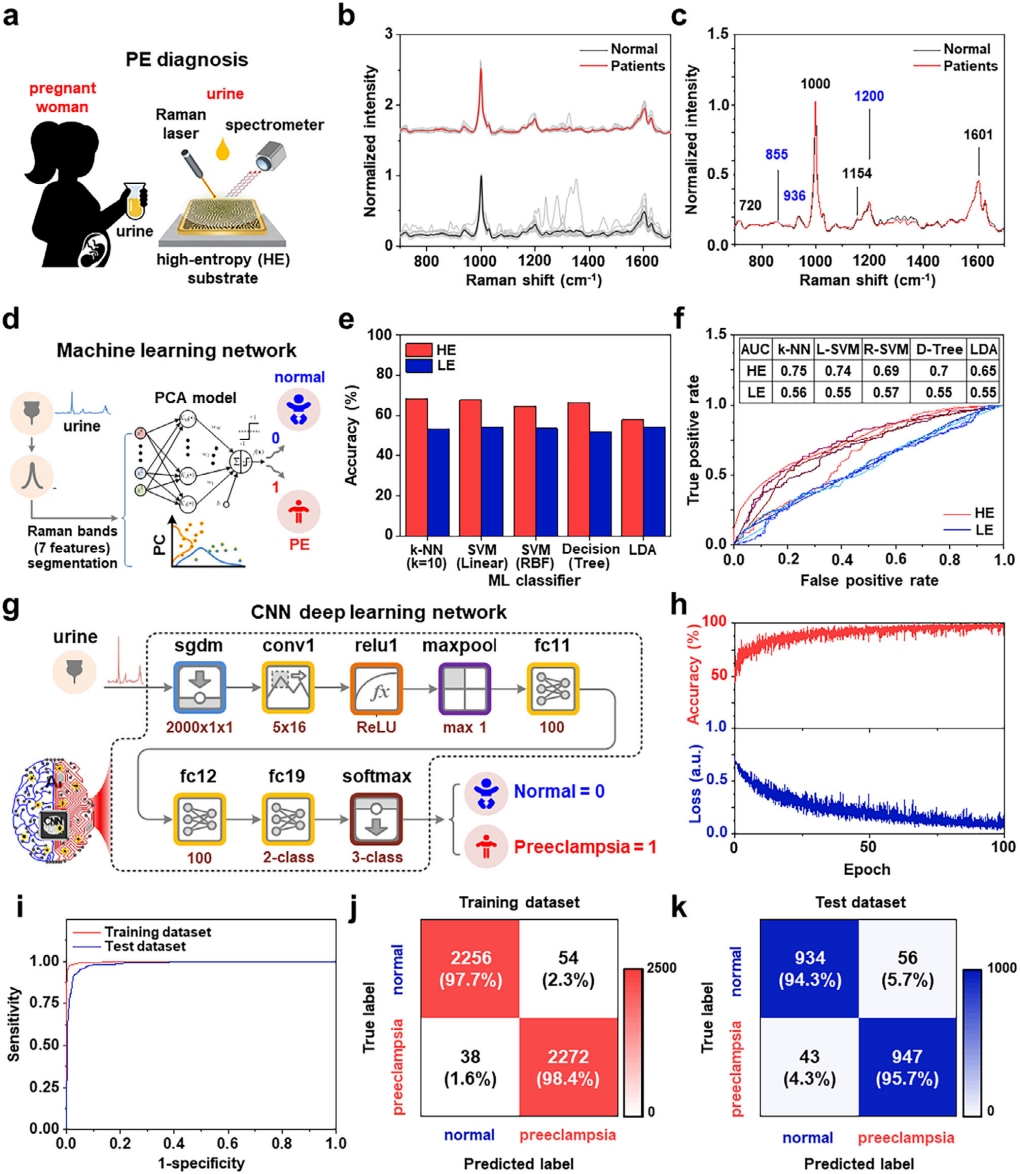
(a) Schematic illustration of the proposed artificial intelligence SERS‐based strategy for early diagnosis of preeclampsia (PE). (b) Normalized averaged SERS spectra of human urine samples acquired using the HE substrate from healthy pregnant controls and PE patients (*n* = 11 per group). (c) Representative Raman peak assignments of human urine samples for the two experimental groups. (d) Schematic illustration of the machine learning (ML) framework for binary classification of healthy controls and PE patients using human urine samples. Performance comparison of five representative classifiers, including *k*‐nearest neighbors (*k*‐NN), support vector machine (SVM) with linear and radial basis function kernels, decision tree, and linear discriminant analysis (LDA), based on principal component analysis (PCA)‐reduced Raman spectral features. Classification performance was evaluated using (e) accuracy and (f) receiver operating characteristic (ROC) curves for the HE and LE substrates. AUC = the area under the curve. (g) Schematic illustration of the deep convolutional neural network (CNN) architecture for binary classification of healthy controls and PE patients using human urine samples. (h) Learning curve and (i) ROC curves of the CNN‐based deep learning classification model trained on the human urine dataset acquired using the HE substrate. Confusion matrices of the proposed CNN‐based deep learning classification model for (j) the training and (k) the test datasets.

Nevertheless, subtle multivariate variations across metabolite‐related peaks can collectively provide discriminatory information beyond single‐band comparisons. Accordingly, seven prominent Raman bands were selected as input features based on their intensity, spectral stability, and relevance to urinary chemistry: four metabolite‐associated bands at 720, 1000, 1154, and 1161 cm^−1^, together with three non‐specific reference bands at 855, 936, and 1200 cm^−1^. These bands are primarily attributed to C─O─C skeletal stretching of sugars, C─O stretching of citrate, and C─C stretching of lipids, respectively.

To perform binary classification between healthy controls and patients with preeclampsia, we first applied principal component analysis (PCA) to the selected Raman features to reduce collinearity and dimensionality. The resulting PCA scores were then used to train five representative classifiers: (i) k‐nearest neighbors (k‐NN), support vector machines (SVMs) with (ii) linear and (iii) radial basis function kernels, (iv) a decision tree, and (v) linear discriminant analysis (LDA) (Figure 4d). To obtain a large dataset, 300 Raman spectra were acquired per subject in each group. Across the classifiers evaluated, HE consistently outperformed LE, achieving a higher mean accuracy (65.1% vs 53.5%). The best‐performing model for HE was the PCA‐reduced 10‐NN classifier (68.5%), while the PCA‐reduced linear SVM classifier (54.4%) yielded the highest accuracy for LE (Figure 4e). Receiver operating characteristic (ROC) analysis further supported the superior discriminative performance of HE (AUC = 0.71) compared with LE (AUC = 0.56) (Figure 4f). Collectively, these results indicate that the HE substrate provides improved performance over the LE substrate for machine‐learning‐assisted SERS sensing. Nevertheless, the modest classification accuracy motivated the adoption of deep learning approaches to further enhance diagnostic performance.

The developed convolutional neural network (CNN) processes a 1D spectral input through convolutional layers with rectified linear unit (ReLU) activation and global max pooling, followed by fully connected (FC) layers and a softmax output for binary classification (Figure 4g). The CNN deep learning model was trained to classify urine SERS spectra from normal controls and patients with preeclampsia, exhibiting stable convergence within 100 training epochs (Figure 4h). Rapid convergence was observed, with the training loss decreasing below 0.1 and the accuracy exceeding 80% within the first 15 epochs, yielding an AUC of 0.9968 in ROC analysis (Figure 4i). The trained CNN‐powered binary classification model generated confusion matrices for both the training (Figure 4j) and test (Figure 4k) datasets. Based on 10‐fold cross‐validation, the proposed deep learning model achieved an average accuracy of 85.3% ± 1.5%, with a sensitivity of 84.7% ± 5.5%, a specificity of 86% ± 5.7%, and AUC of 0.9327 ± 0.0122 (Table S2). The corresponding false discovery rates (FDRs) were 2.6% for the training dataset and 13.8% for the test dataset. Collectively, these results support the feasibility of combining the HE substrate with AI‐driven analysis for noninvasive, urine‐based discrimination of preeclampsia, including samples collected before 20 weeks of gestation. This proof‐of‐concept highlights the potential of data‐driven SERS diagnostics for early screening, warranting larger‐scale clinical validation.

## 3 Conclusion

In conclusion, we developed a grain‐size‐engineered lamellar BCP platform that introduces controlled local orientational disorder while preserving nanoscale periodicity, thereby enabling a reproducible and polarization‐robust SERS substrate. Under optimized conditions, HE delivered stronger SERS signals with markedly reduced spatial and angular variability, as supported by mapping, rotation measurements, and wafer‐scale demonstrations. Building on this large‐area uniformity, we demonstrated the feasibility of integrating HE substrates with data‐driven analysis to discriminate preeclampsia using urine samples collected before 20 weeks of gestation. PCA‐based classifiers showed modest performance, whereas a 1D CNN substantially improved classification metrics, highlighting the advantage of deep learning for capturing subtle multivariate spectral signatures. Together, these results establish a scalable route to isotropic, reproducible SERS substrates compatible with AI‐driven diagnostics and motivate further subject‐level validation in larger, independent cohorts to assess generalizability for early screening.

## 4 Experimental Section

### 4.1 Materials

All the polymers, including BCPs and hydroxyl‐terminated PS‐*r*‐PMMA random copolymers, were purchased from Polymer Source Inc. All bulk solvents, thiophenol (97%), and 4‐NTP (80%) were purchased from Sigma–Aldrich.

### 4.2 Preparation of Block Copolymer Nanopatterned Films

The BCP and hydroxyl‐terminated random copolymers were used without further purification. PS‐*r*‐PMMA brush thin films were spin‐coated on UV‐ozonated silicon substrates with a 1 wt% toluene solution, followed by annealing at 160°C for 12 h under vacuum to obtain a neutral surface. The substrates were then rinsed with toluene to remove unreacted random copolymer brushes, and PS‐b‐PMMA (Mn: 105 kg mol^−^^1^‐b‐106 kg mol^−^^1^) thin films were spin‐coated onto the brush‐treated silicon substrates from a 1.5 wt% toluene solution. The BCP thin films were annealed at room temperature in a tetrahydrofuran (THF) vapor atmosphere for 1–2 h, followed by annealing at 250°C for 4 h.

### 4.3 Au Nanogap Array Formation

To obtain sharp PS posts and prevent pattern collapse, the PS domains were crosslinked with UV irradiation. The PMMA matrix of the self‐assembled BCP thin film was selectively etched with O_2_/Ar plasma reactive ion etching (RIE) to leave only the PS posts. Au thin film was e‐beam evaporated onto the entire substrate surface with a deposition thickness of 10–60 nm.

### 4.4 Preparation of Samples for Raman Spectroscopy

Au nanogap arrays were immersed in a 1 mM thiophenol solution in ethanol for 12 h under ambient conditions. Then, the samples were washed several times with ethanol and dried under the gentle blowing of nitrogen gas.

### 4.5 Characterization

The periodicity of lamellar patterns by grain size was confirmed via Grazing‐Incidence Small‐Angle X‐ray Scattering (GI‐SAXS, Rigaku Nanopix). Top and cross‐sectional morphologies of BCP nanostructures and Au nanoplasmonic arrays were characterized using a Scanning Electron Microscopy (SEM, Hitachi S‐4800) and Transmission Electron Microscopy (TEM, JEOL JEM‐ARM200F). Raman spectra of thiophenol molecules on nanogap arrays were acquired using a high‐resolution dispersive Raman spectrometer (LabRAM HR‐800, Horiba) and a 633 nm laser. Raman spectra were collected 10 times for 10 seconds for all samples (Beam diameter: 0.84 µm).

The SERS performance of the optimized substrates at different Raman excitation wavelengths was evaluated using a multiwavelength Raman spectroscopy system (Uni‐DRON, UniNanoTech, Korea). Three representative excitation lasers (532, 638, and 785 nm) were employed with a 50× objective lens (numerical aperture = 0.8). The substrates were immersed in 4‐NTP solutions of varying concentrations and biofluids for 30 min, followed by drying under ambient conditions. SERS spectra were acquired from 10 × 10 mapping points in three randomly selected areas under the following conditions: Raman shift range of 400–1800 cm^−1^, spectral resolution below 1 cm^−1^, acquisition time of 1 s, and laser powers of 4.0 mW (532 nm), 6.5 mW (638 nm), and 7.2 mW (785 nm). Each laser power was measured at the objective lens tip using a handheld power meter (PM160, Thorlabs, USA). Baseline correction was applied to the SERS spectra, while the unprocessed spectra were used for deep learning analysis.

Baseline correction was performed on the SERS spectra using the Adaptive Iteratively Reweighted Penalized Least Squares (airPLS) algorithm implemented in Peak Spectroscopy Software (Operant LLC, USA), while the unprocessed spectra were used for deep learning analysis. All data were expressed as mean ± standard deviation.

### 4.6 Orientation Correlation Length Analysis

Local orientation maps were generated using structure tensor analysis of Gaussian‐smoothed (σ = 3 pixels) grayscale images. The two‐point orientational correlation function was defined as 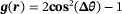 and evaluated via Monte Carlo sampling (main analysis: 5 × 10^5^ pairs; preliminary scan: 2 × 10^5^ pairs). Here, **Δ** ***θ*** accounts for nematic symmetry, and distances ***r*** were radially binned at 1‐pixel intervals.

Image‐specific optimal maximum analysis distances ***r***_**max**_ were automatically determined via preliminary scans. The first point ***r***_end_ where ***g*** (***r***) falls below a noise floor of 0.1 was identified, followed by setting ***r***_**max**_ = * *1.5 × ***r***_end_ (minimum 50 pixels). This adaptive approach excludes noise‐dominated regions while capturing the full decay curve.

The positive ***g*** (***r***) region was fitted to the exponential decay model g(r)=exp(−r/ξ) to extract the correlation length ***ξ*** (in nm). Pixel‐to‐nm conversion used 2.48 nm/pixel from the microscope scale bar.

### 4.7 Orientational Entropy Analysis

Grayscale images were loaded, normalized to, and mean‐subtracted prior to 2D fast Fourier transform (FFT). The power spectrum ∣ ***FFT*** ∣^2^ was computed and radially averaged to identify the peak radial frequency ***q*** ^*^ corresponding to the dominant structural periodicity (low‐frequency cutoff applied to exclude DC noise).

An annular “donut” region was defined around ***q*** ^*^ (±5 pixels), and the azimuthal intensity profile was extracted by angular binning (1° bins, 0°–180° range accounting for directional symmetry). The normalized profile intensities ***p***_***i***_ were used to compute the Shannon entropy

(1)

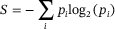

where ***S*** quantifies the directional disorder (low ***S***: anisotropic alignment; high ***S***: isotropic distribution).

### 4.8 Interfacial Length Density Analysis

Grayscale images were normalized to and analyzed via 2D FFT to measure the lamellar period from the peak radial frequency ***q*** ^*^ in the log‐scaled power spectrum (low‐frequency cutoff = 5 pixels). A correction factor was computed as ***P***_measured_/***P***_standard_ (standard = 25 pixels) for period‐normalized interfacial length across samples. Noise reduction was achieved with Gaussian blurring (5×5 kernel), followed by Otsu thresholding for automatic phase binarization. Raw interfacial length was calculated by counting horizontal and vertical pixel transitions:

(2) $$L_{\mathrm{raw}}=N_{\mathrm{horizontal}}+N_{\mathrm{vertical}}$$

with corrected length ***L***_corrected_ = ***L***_raw_** × (***P***_measured_/***P***_standard_) and conversion to nm using 2.48 nm/pixel. Interfacial density was computed as ***D *** = ***L***_corrected_ /***A***, where ***A*** is the physical image area in nm^2^ (width × height in nm).

### 4.9 Limit of Detection and SERS Enhancement Factor Calculation

The SERS intensity under 785 nm excitation exhibited a linear dependence on 4‐NTP concentration from 10^−8^ to 10^−5^ M (R^2^ = 0.989), followed by saturation at higher concentrations (Figure S5), consistent with adsorption approaching surface coverage after rinsing removes unbound molecules. The baseline signal in the absence of analyte was ∼100, and the limit of detection (LOD) was estimated to be 67.3 nM based on an SNR > 3 criterion using the linear fit. The corresponding EF was ∼2.1 × 10^5^ (Figure S6), demonstrating sufficient sensitivity for low‐concentration detection. The average SERS enhancement factor (EF) of the optimized substrate was calculated from the formula

(3) $$\mathrm{EF}=\frac{I_{\mathrm{SERS}}/N_{\mathrm{SERS}}}{I_{\mathrm{ref}}/N_{\mathrm{ref}}}$$

where *I_SERS_* was the measured SERS intensity, *N_SERS_* was the number of molecules bound to the enhancing SERS‐active structure, *I*_ref_ was the measured Raman intensity without enhancement, and *N*_ref_ was the number of molecules in the excitation volume for measuring *I*_ref_.

### 4.10 Numerical Analysis

The electric field distributions and polarization‐dependent optical responses of the substrates were analyzed using finite element analysis (FEA) in the RF Module of COMSOL Multiphysics. 2D models of BCP lamellar patterns were constructed from SEM images to represent top‐view and cross‐sectional geometries. The nanogap width in the cross‐sectional model was set based on the TEM‐derived average gap width of 8.5 ± 1.56 nm obtained from Figure 1h. Polarization‐dependent electric field responses were evaluated by rotating the incident polarization angle from 0° to 180° while monitoring the maximum local electric field intensity at excitation wavelengths of 532, 638, and 785 nm. Material parameters were taken from the literature [62], and perfectly matched layers (PMLs) were applied at the outer boundaries of the computational domain to suppress artificial reflections (Table S3). The PML thickness was set to 200 nm, corresponding to approximately one‐quarter of the longest excitation wavelength (785 nm). The distance between the Au nanostructure and the PML boundary was maintained at least 300 nm to prevent interactions between the localized plasmonic field and the absorbing boundary.

### 4.11 AI‐Based Analysis

A total of 6600 SERS spectra were randomly divided into training (70%, 4,620 spectra) and test (30%, 1980 spectra) datasets. All machine learning and deep learning analyses were implemented using MATLAB (MathWorks, USA) [63]. Machine learning‐based classification was performed to distinguish urine samples from healthy controls and patients with preeclampsia. Seven representative Raman bands were selected as input features, and PCA was applied for dimensionality reduction. Five supervised classifiers were evaluated, and performance was assessed using accuracy and ROC analysis, with AUC as the primary metric. A CNN‐based deep learning model was further developed to improve classification performance. The CNN directly processed 1D SERS spectra (210–2,030 cm^−1^) using 1D convolutional layers with ReLU activation and global max pooling, followed by FC layers and a softmax output for binary classification. The model was trained for 100 epochs, and its performance was evaluated by 10‐fold cross‐validation in terms of accuracy, sensitivity, specificity, and AUC. Confusion matrices and FDRs were also analyzed.

### 4.12 Urine Collection

Patients were recruited from ward or delivery suite admissions at Uijeongbu St. Mary's Hospital (Gyeonggi‐do, Korea) and provided written informed consent prior to urine collection. The study was approved by the Institutional Review Board of the Catholic Medical Center of the Catholic University of Korea (UC24ZISI0257). Participants (n = 11 per group) were classified into a control group of normotensive pregnant women without proteinuria or preeclampsia‐related complications and a preeclampsia group diagnosed according to the American College of Obstetricians and Gynecologists guidelines [64]. Preeclampsia was defined as new‐onset hypertension after 20 weeks of gestation, with proteinuria or, in its absence, hypertension accompanied by signs of multi‐organ dysfunction. Maternal clinical data were collected, and urine samples were stored at −70°C.

### 4.13 Statistical Analysis

Data are presented as mean ± standard deviation (SD) unless otherwise specified. Relative standard deviation (RSD) was calculated as the SD divided by the mean and expressed as a percentage. For structural and SERS reproducibility analyses, the number of measured data points is specified according to each experiment. The nanogap width distribution was obtained from 100 measured gap positions in cross‐sectional TEM images. Local SERS mapping reproducibility was evaluated from 121 measurement points within a 50 µm × 50 µm area, and wafer‐scale spot‐to‐spot reproducibility was evaluated from 15 measurement positions across a 4‐inch wafer‐scale substrate.

For urine SERS analysis, biological samples were collected from healthy pregnant controls and patients with preeclampsia (n = 11 per group). A total of 300 SERS spectra were acquired from each subject, yielding 6600 spectra in total. Univariate comparisons of selected Raman band intensities between the two groups were performed to evaluate single‐band differences in protein‐associated spectral regions.

For AI‐based classification, the full spectral dataset was split into training (70%, 4620 spectra) and test (30%, 1980 spectra) sets. Classification performance was quantified using accuracy, sensitivity, specificity, AUC, and FDR; no inferential significance threshold was applied, as model evaluation relied on cross‐validation‐based performance metrics. All analyses were implemented in MATLAB (MathWorks, USA; Section 4.11).

## Author Contributions

**Wonsik Kim**: methodology, Writing – original draft, data curation, investigation, visualization, validation. **Jin Man Kim**: methodology, writing – original draft, data curation, validation, formal analysis, investigation, visualization, conceptualization. **Wansun Kim**: validation, software, visualization, investigation, writing – original draft, formal analysis, data curation, methodology. **Jang Hwan Kim**: writing – review and editing, project administration, supervision, conceptualization, data curation, resources, funding acquisition. **Hyeong Min Jin**: conceptualization, supervision, writing – review and editing, funding acquisition, project administration, resources, data curation. **Samjin Choi**: writing – review and editing, supervision, software, validation, resources, data curation. **Yeon–Hee Kim**: writing – review and editing, supervision, data curation, formal analysis, resources.

## Conflicts of Interest

The authors declare no conflicts of interest.

## Supporting information




**Supporting File**: advs76631‐sup‐0001‐SuppMat.docx.

## Data Availability

The data that support the findings of this study are available from the corresponding author upon reasonable request.
