# Supplementary material for: Maximizing Nanoscale Disorder in Block Copolymers for Orientation‐Independent SERS Platform Toward Non‐Invasive Diagnostics
Source: Adv Sci (Weinh). 2026 Jul 14:e76631. Online ahead of print. doi: 10.1002/advs.76631 (PMC13367102; doi:10.1002/advs.76631)
Supplement: Supplementary file 1 — Supporting File: advs76631‐sup‐0001‐SuppMat.docx. [file ADVS-9999-e76631-s001.docx]

Supporting Information

Maximizing Nanoscale Disorder in Block Copolymers for Orientation-Independent SERS Platform toward Non-Invasive Diagnostics

Jin Man Kim^1,2‡^, Wonsik Kim^3‡^, Wansun Kim^4‡^, Yeon-Hee Kim^5*^, Samjin Choi^4*^,
Jang Hwan Kim^3,6*^ and Hyeong Min Jin^1,2*^

*^1^ Department of Organic Materials Engineering, Chungnam National University, Daejeon 34134, Republic of Korea*

*^2^ Department of Materials Science and Engineering, Chungnam National University, Daejeon, 34134, Republic of Korea*

*^3^ Department of Energy Systems Research, Ajou University, Suwon, 16499, Republic of Korea*

*^4^ Department of Medical Engineering, Graduate School, Kyung Hee University, Seoul 02447, Republic of Korea*

*^5^ Department of Obstetrics & Gynecology, Uijeongbu St. Mary‘s Hospital, The Catholic University of Korea College of Medicine, Gyeonggi-do, Republic of Korea*

*^6^ Department of Materials Science and Engineering, Ajou University, Suwon 16499, Republic of Korea*

** Corresponding authors.
E-mail addresses:* hyeongmin@cnu.ac.kr (H.M.Jin), janghwankim@ajou.ac.kr (J.H.Kim), medchoi@khu.ac.kr (S.Choi), yoni@catholic.ac.kr (Y.-H.Kim)

^‡^ *These authors contributed equally to this work*

**This file includes:**

Figures S1 to S7

Table S1 to S3

**
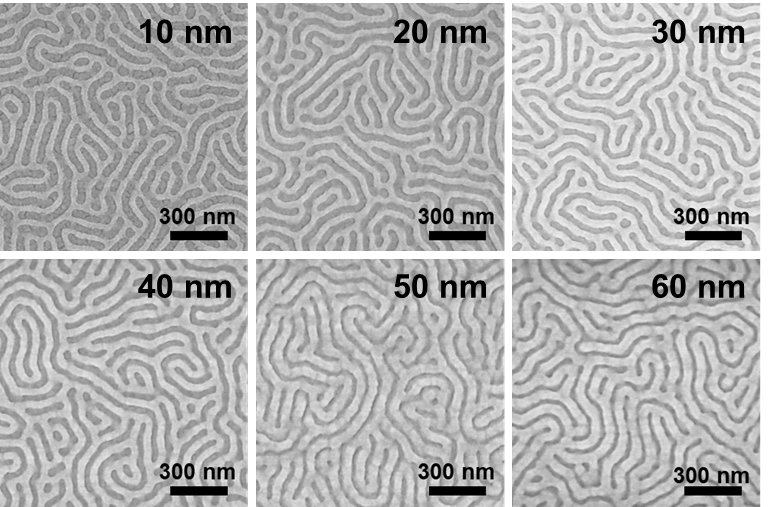
**

**Figure S1.** Top-view SEM images of the HE as a function of Au deposition thickness.

**
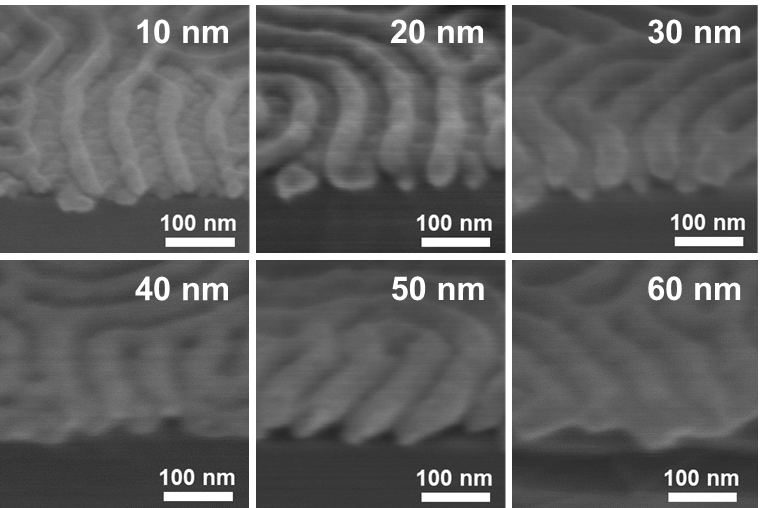
**

**Figure S2.** Tilt-view SEM images of the HE as a function of Au deposition thickness.

**
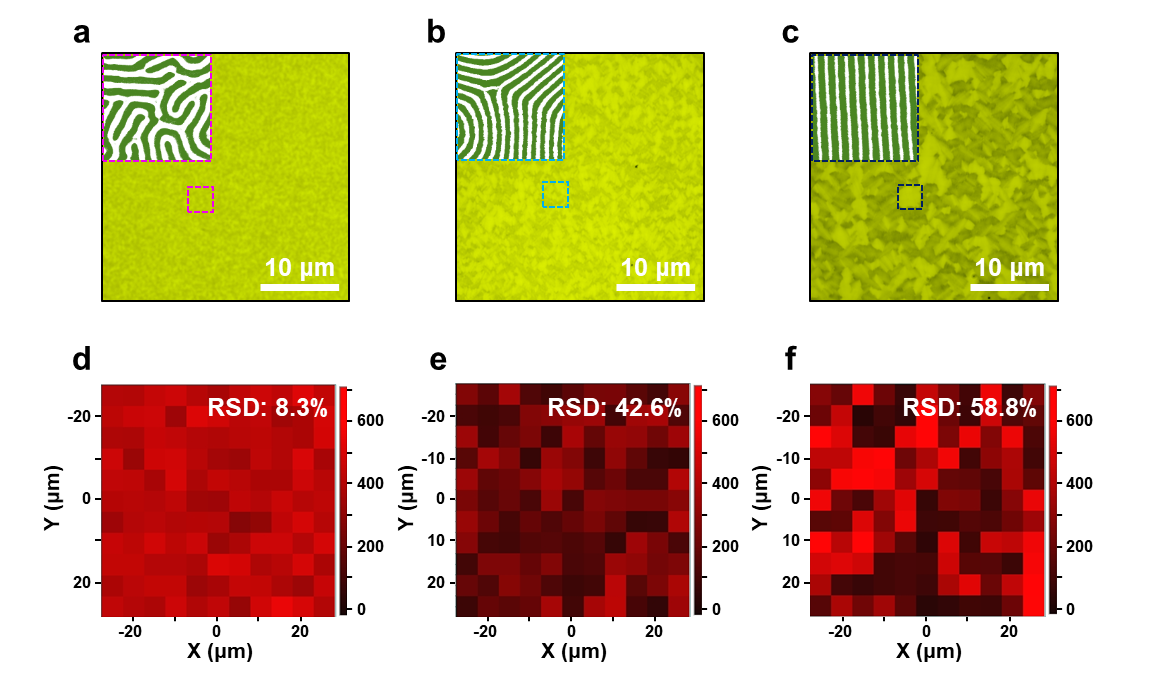
**

**Figure S3.** (a-c) Polarized optical microscopy (POM) images of HE, ME, and LE. (d-f) Raman intensity maps of HE, ME, and LE over a 50 μm × 50 μm area.

**
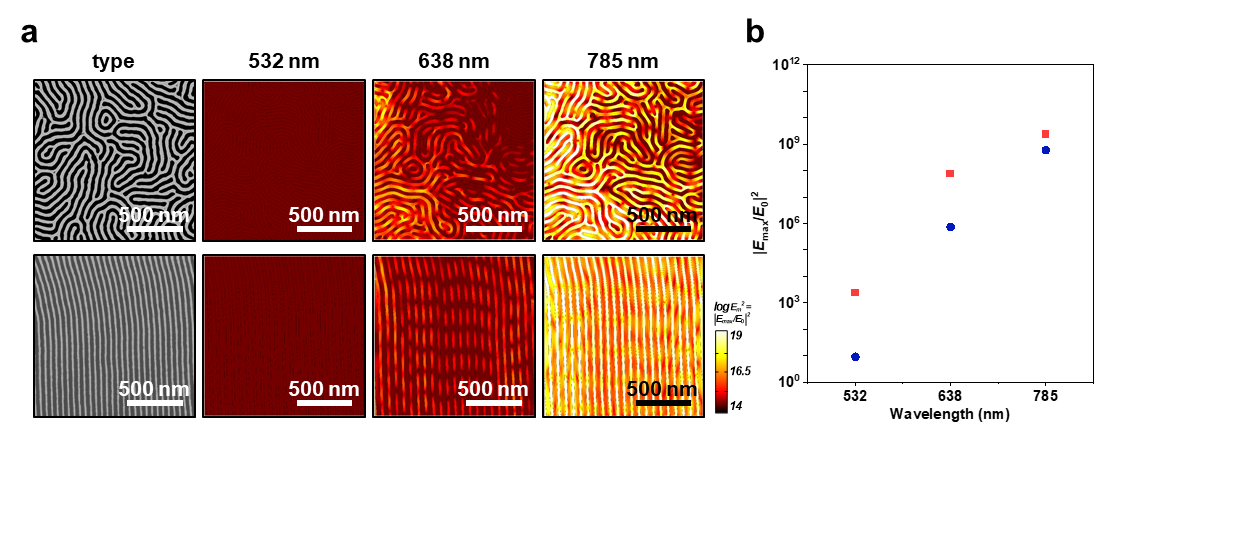
**

**Figure S4**. (a) FEA-simulated top-view electric field distributions and (b) comparative analysis of electric field enhancements for HE and LE substrate models at three different excitation wavelengths.

**Figure S5**. Linear calibration curve and determination of limit of detection (LOD) for the substrate using different concentrations of 4-NTP.

**Figure S6**. Raman spectrum of 10 mM 4-NTP and the SERS spectrum of 100 nM 4-NTP measured on a 50 nm-thick gold film and the HE substrate, respectively.


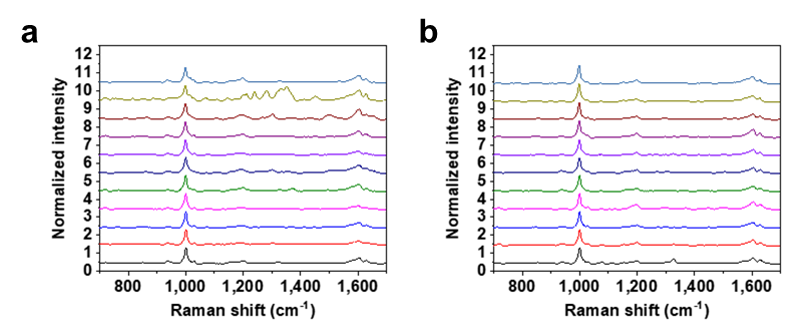


**Figure S7**. Variations in the SERS spectra of urine samples collected from (a) healthy pregnant controls and (b) PE patients (*n* = 11 per group), acquired using the proposed HE substrate.

**Table S1.** Comparison of enhancement factor (EF), signal reproducibility (RSD), and fabrication characteristics of representative state-of-the-art SERS substrates.

| **Substrate/structure** | **Fabrication method** | **EF** | **RSD (%)** | **Scale** | **Polarization- independent** | **Ref.** |
| --- | --- | --- | --- | --- | --- | --- |
| ALD-defined triangular Au nanogap (sub-10 nm) | Colloidal lithography + ALD | ~10⁸ (10⁹ at 5 nm) | N/R | Wafer | No | [54] |
| Photolithography/swelling-crack Au nanogap | Photolithography + sputtering | ~10⁸ | N/R | Wafer | No | [55] |
| Annular Au nanogap array (10 nm) | NSL + adhesion lithography | ~10⁷ | < 10 | cm² | Yes | [56] |
| Laser-MBE Ag nanoislands (~5 nm gap) | Laser molecular beam epitaxy | ~1.2 × 10⁵ | 7.8 | 6×6 cm² | N/R | [57] |
| Cicada-wing-templated Ag/PDMS (flexible) | Magnetron sputtering replica | ~4.2 × 10⁵ | 2.8–7.3 | cm² | Yes | [58] |
| BCP-nanopost Au nanogap array | BCP self-assembly + e-beam | ~10⁴ | 12.3 | 2-inch | Yes | [19] |
| ***HE BCP lamellar nanogap (this work)*** | BCP self-assembly + e-beam | ~2.1 × 10⁵ | 7.2 | 4-inch | Yes | – |

**Table S2.** Performance of CNN-based deep learning classification model for early diagnosis of PE using 10-fold cross-validation.

| K-fold Cross-Validation Results – Training Dataset | | | | | | | | | |
| --- | --- | --- | --- | --- | --- | --- | --- | --- | --- |
| K | TP | FP | FN | TN | SEN | SPE | ACC | FDR | AUC |
| 1 | 2885 | 75 | 56 | 2924 | 98.1 | 97.5 | 97.79 | 2.53 | 0.9963 |
| 2 | 2660 | 12 | 325 | 2943 | 89.1 | 99.59 | 94.33 | 0.45 | 0.9972 |
| 3 | 2886 | 19 | 92 | 2943 | 96.91 | 99.36 | 98.13 | 0.65 | 0.998 |
| 4 | 2779 | 31 | 175 | 2955 | 94.08 | 98.96 | 96.53 | 1.1 | 0.9952 |
| 5 | 2859 | 66 | 122 | 2893 | 95.91 | 97.77 | 96.84 | 2.26 | 0.9954 |
| 6 | 2961 | 138 | 23 | 2818 | 99.23 | 95.33 | 97.29 | 4.45 | 0.9975 |
| 7 | 2973 | 257 | 5 | 2705 | 99.83 | 91.32 | 95.59 | 7.96 | 0.9992 |
| 8 | 2908 | 64 | 49 | 2919 | 98.34 | 97.85 | 98.1 | 2.15 | 0.9974 |
| 9 | 2885 | 123 | 102 | 2830 | 96.59 | 95.83 | 96.21 | 4.09 | 0.9926 |
| 10 | 2806 | 4 | 149 | 2981 | 94.96 | 99.87 | 97.42 | 0.14 | 0.9994 |
| mean | 2860 | 79 | 110 | 2891 | 96.3 | 97.34 | 96.82 | 2.58 | 0.9968 |

| K-fold Cross-Validation Results – Test Dataset | | | | | | | | | |
| --- | --- | --- | --- | --- | --- | --- | --- | --- | --- |
| K | TP | FP | FN | TN | SEN | SPE | ACC | FDR | AUC |
| 1 | 310 | 43 | 49 | 258 | 86.35 | 85.71 | 86.06 | 12.18 | 0.9363 |
| 2 | 244 | 23 | 71 | 322 | 77.46 | 93.33 | 85.76 | 8.61 | 0.9503 |
| 3 | 273 | 39 | 49 | 299 | 84.78 | 88.46 | 86.67 | 12.5 | 0.9393 |
| 4 | 270 | 38 | 76 | 276 | 78.03 | 87.9 | 82.73 | 12.34 | 0.9132 |
| 5 | 268 | 36 | 51 | 305 | 84.01 | 89.44 | 86.82 | 11.84 | 0.9284 |
| 6 | 288 | 56 | 28 | 288 | 91.14 | 83.72 | 87.27 | 16.28 | 0.945 |
| 7 | 303 | 91 | 19 | 247 | 94.1 | 73.08 | 83.33 | 23.1 | 0.9239 |
| 8 | 291 | 46 | 52 | 271 | 84.84 | 85.49 | 85.15 | 13.65 | 0.9176 |
| 9 | 274 | 63 | 39 | 284 | 87.54 | 81.84 | 84.55 | 18.69 | 0.9305 |
| 10 | 273 | 27 | 72 | 288 | 79.13 | 91.43 | 85.0 | 9.0 | 0.9429 |
| mean | 279 | 46 | 51 | 284 | 84.67 | 86.0 | 85.33 | 13.82 | 0.9327 |

**Table S3.** Specifications of finite element models including material optical constants and mesh properties.

| Wavelength-Dependent Optical Constants of Materials used in FEA Simulations | | |
| --- | --- | --- |
| Material | Refractive Index (*n*) | Extinction Coefficient (*k*) |
| Air | 1.00 | 0.00 |
| Au (532 nm) | 0.52 | 2.37 |
| Au (638 nm) | 0.16 | 3.42 |
| Au (785 nm) | 0.17 | 4.89 |
| PS (532 nm) | 1.60 | 0.00 |
| PS (638 nm) | 1.59 | 0.00 |
| PS (785 nm) | 1.58 | 0.00 |

| FEA Model Specifications for BCP Templates | | | | |
| --- | --- | --- | --- | --- |
|  | Cross-Sectional View | | Top-View | |
|  | HE | LE | HE | LE |
| Mesh Type | Triangular elements | | | |
| Mesh Size | 0.2–50 nm | | | |
| Average Element Quality | 0.8322 | 0.8418 | 0.8265 | 0.8252 |
| Number of Elements | 67,451 | 88,758 | 223,890 | 103,356 |
| Number of DOFs | 337,770 | 444,306 | 1,119,762 | 517,092 |
